# Changes in Liver Gene Expression and Plasma Concentration of Rbp4, Fetuin-A, and Fgf21 in Sprague-Dawley Rats Subjected to Different Dietary Interventions and Bariatric Surgery

**DOI:** 10.1155/2018/3472190

**Published:** 2018-08-16

**Authors:** Dominika Stygar, Wojciech Pigłowski, Elżbieta Chełmecka, Bronisława Skrzep-Poloczek, Tomasz Sawczyn, Wojciech Garłowski, Jerzy Jochem, Konrad Wojciech Karcz

**Affiliations:** ^1^Department of Physiology, School of Medicine with Dentistry Division in Zabrze, Medical University of Silesia, Katowice, Poland; ^2^Center for Translational Research and Molecular Biology of Cancer, Maria Skłodowska-Curie Institute, Oncology Center, Gliwice, Poland; ^3^Tumor Pathology Department, Maria Skłodowska-Curie Institute, Oncology Center, Gliwice, Poland; ^4^Department of Statistics, School of Pharmacy with the Division of Laboratory Medicine in Sosnowiec, Medical University of Silesia, Katowice, Poland; ^5^Clinic of General, Visceral, Transplantation and Vascular Surgery, Hospital of the Ludwig Maximilian University, Munich, Germany

## Abstract

**Purpose:**

To study the effect of duodenal-jejunal omega switch (DJOS) in combination with different dietary patterns on the retinol-binding protein (RBP4), fetuin-A, and fibroblast growth factor 21 (FGF21) plasma levels and their hepatic gene expressions in rats.

**Methods:**

A high-fat diet (HF) was given to 28 rats and 28 more were fed with a control diet (CD) for 2 months. After that, half of each group underwent either DJOS or SHAM surgery. For the next 2 months, half of the animals in each operation group were kept on the same diet as before and half of them had the diet changed. After 16 weeks of the experiment RBP4, fetuin-A, and FGF21 plasma levels as well as liver* Rbp4*,* Ahsg*, and* Fgf21* gene expressions were measured.

**Results:**

DJOS had a reductive impact on plasma levels of RBP4, fetuin-A, and FGF21 and* Rbp4*,* Ahsg, *and* Fgf21* relative gene expression in the liver when compared to SHAM. The HF/HF group expressed significantly higher RBP4 and fetuin-A plasma levels in comparison to the control. The HF diet used before and/or after surgery led to upregulation of* Rbp4*,* Ahsg*, and* Fgf21* relative gene expression. The lowest levels of analyzed parameters were observed in the CD/CD group.

**Conclusions:**

The efficiency of DJOS surgery, measured by hepatokines' plasma levels and their gene expressions in the liver, depends on the type of diet applied before and after surgery. Manipulation of dietary patterns can lead to marked improvements in metabolic profile after DJOS surgery.

## 1. Introduction

Overconsumption of any form of dietary energy leads to overweight, obesity, diabetes mellitus type 2 (T2DM), and related comorbidities [1–3].

The studies of the effect of metabolic surgery focus broadly on the general health improvement [4]. A retrospective cohort study of patients with T2DM who had undergone RYGB surgery showed that 70% of the patients experienced a complete remission in the first 5 years, but among these 35% developed T2DM within 5 years after remission. Secretory proteins released from the liver, called hepatokines, are related to the development of impaired glucose and lipid metabolism. RBP4 acts as a negative acute phase inflammatory protein and contributes to systematic insulin resistance; it is upregulated in patients with obesity and T2DM [5]. Enhanced expression of the* Rpb4 *gene is correlated with increased risk of T2DM and may be one of triggering factors for the disease development [6]. Plasma retinol-binding protein (RBP4) and its overexpression is a biomarker for insulin resistance, prediabetes and T2DM, the metabolic syndrome, and myocardial infarction [7–10]. Fetuin-A enhances lipid accumulation and activates inflammatory signalling in the liver. This protein is involved in the regulation of postprandial glucose disposal, insulin sensitivity, weight gain, and fat accumulation [11]. A significant increase in fetuin-A (*Ahsg*) gene expression has been observed in an animal diet-induced obesity model [12]. Finally, the hepatic fibroblast growth factor 21 (FGF21) enhances lipid oxidation and lipolysis, suppresses hepatic steatosis, and decreases obesity-induced insulin resistance [11].

DJOS is a type of bariatric surgery, with proximal loop duodenoenterostomy, which bypasses the foregut and directly stimulates the hindgut [13]. The advantage of DJOS is that the pylorus of the patients stays intact, which reduces symptoms characteristic for postgastrectomy conditions such as dumping, diarrhoea, and dyspepsia [14, 15]. DJOS is a relatively new technique; thus an animal model, for exploring the physiological, long-term effects of this procedure, is still needed [14–16]. The aim of this work was to study the efficiency of duodenal-jejunal omega switch (DJOS) in relation to different dietary patterns, measured by the retinol-binding protein (RBP4), fetuin-A, and fibroblast growth factor 21 (FGF21) plasma levels and their hepatic gene expressions in rats. Nutrient rich diet and bariatric surgery may strongly stimulate hepatokines' metabolic pathways, levels, and activity [17, 18]. Thus, the diet combinations used were intended to imitate various dietary behaviours of patients, both before and after the surgery. As the liver plays a major role in the regulation of systemic glucose metabolism, which may result in impaired systemic glycaemic control and the development of T2DM, we focused on liver function and measured the liver gene expression of* Rbp4, Ahsg, *and* Fgf21* [17]. We studied applied dietary patterns and selected hepatokines in relation to duodenal exclusion surgery, which is becoming one of the popular bariatric procedures. Finally, we studied the ability of different dietary patterns to influence RBP4, fetuin-A, and FGF21 plasma levels and their liver gene expressions.

## 2. Materials and Methods

### 2.1. Animals

Seven-week-old male Sprague-Dawley rats were acquired from Charles River Breeding Laboratories (Wilmington, Mass.). Animal care was performed according to previously described procedures [19]. Animals were kept in with 12 h light-dark cycles at 22°C and 40–60% humidity. Environmental enrichment was provided, and all rats had free access to water and food. The composition of control diet (CD) was as follows: 24% protein, 4.9% fat, 7% crude ashes, and 4.7% crude fiber (Provimi Kliba AG, Kaiseraugst, Switzerland). The animals from control group were kept on CD for the period of two months, before and after surgery. Obesity was induced by keeping the animals on a high-fat diet (HF; 23.0 kJ/g; 59% fat, 27% carbohydrate, and 14% protein (Ssniff Spezialdiäten GmbH, Soest, Germany)) for the period of two months before and after surgery. Full description of diets used in the experiment is presented in the supplementary materials (supplementary Table 1). All rats fasted overnight before surgery. All experimental procedures were approved by the Local Ethics Committee (58/2014). All applicable institutional and/or national guidelines for the care and use of animals were followed (EU Directive 2010/63/EU).

### 2.2. Experimental Design

**Figure 1:**
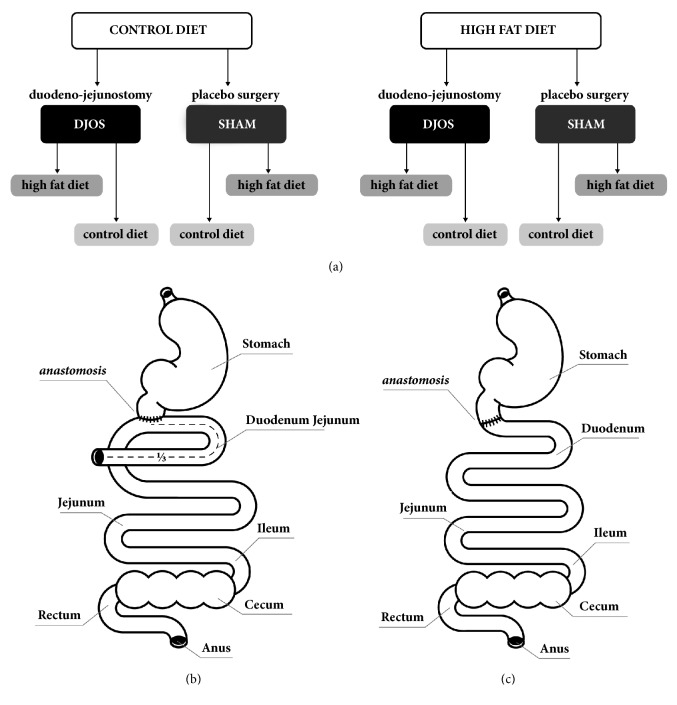
(a) Scheme of experimental groups. (b) Schematic illustrations of duodenal-jejunal omega switch (DJOS) and (c) SHAM surgery.

The experimental design of the study was conducted accordingly to previously reported methodology [19]. Briefly, two types of diet, control diet (CD) and high-fat diet (HF), were applied before surgery for 8 weeks (Figure 1(a)). After surgery, half of the animals from the CD and HF groups had the diet changed (Figure 1(a)). The number of animals included in the study was kept as small as possible in consideration of the “3Rs” for the humane treatment of animals [20]. The numbers of rats that survived till the end of the experiment in the experimental groups were 7, except for the HF/SHAM/CD where 6 rats survived.

A DJOS was performed according to Karcz et al. methodology [16] previously described [19]. Rats were fasted overnight before the surgery. Surgeries were performed under general anaesthesia with 2% isoflurane (AbbVie Deutschland GmbH & Co. KG, Ludwigshafen, Germany) and oxygen flow at 2 l/min under spontaneous breathing. Analgesia was performed with xylazine (5 mg/kg b.w., ip; Xylapan, Vetoquinol Biovet, Poland) and antibiotic prophylaxis with gentamicin (5mg/kg b.w., im). The DJOS and SHAM surgeries have been previously described [19]. After a midline incision of 3–4 cm to gain abdominal access, the total length of the small intestine was measured. The duodenoenterostomy was located approximately at 30% of the total small bowel length (Figure 1(b)). The earlier defined jejunum was anastomosed by end-to-side duodenoenterostomy in order to restore continuity, excluding the duodenum and parts of the small intestine bowel. The stump of the duodenum was closed with cross suture (Prolene 6/0, Ethicon). For the SHAM surgery, transections and reanastomosis were made and closed using PDS 6/0 (Ethicon) in the duodenum and small intestine (Figure 1(c)). Fascia and skin closure were performed using Monocryl 4/0 and Vicryl 4/0.

### 2.3. Tissue Collection and Assays Identification

Before surgery, reference biopsies of liver tissue were collected, rinsed with PBS, and placed into RNAlater RNA Stabilization Reagent (Qiagen, Hilden, Germany). The samples were subsequently snap frozen to –80°C and kept at this temperature for 3 months until* Rbp4*,* Ahsg*,* Fgf21* gene expression analyses were performed.

Eight weeks after surgery, blood for hepatokines' analysis was collected from the abdominal aorta into tubes containing 10 *μ*l of EDTA (Sigma-Aldrich, St. Louis, Mo, USA). After centrifugation at 4 000 rpm for 10 minutes at 4°C, plasma samples were collected and snap frozen in liquid nitrogen and stored at – 80°C until analysis was performed. Hepatokines, such as RBP4, fetuin-A, and FGF21, were assessed in duplicate by ELISA kits (Cloud-Clone Corp., Katy, Tex., USA).

After blood sampling the tissues were harvested and the animals were euthanized. Liver tissue was explanted, rinsed with PBS, and placed into RNAlater RNA Stabilization Reagent (Qiagen, Hilden, Germany). Then samples were snap frozen at –80°C for 1 month until further analysis.

### 2.4. Gene Expression

The liver tissues were taken from animals of all the experimental groups before (control tissue) and after DJOS and SHAM surgery (test tissue). The tissues were homogenised (Lysing Matrix D 1,4 mm ceramic spheres, FastPrep®-24 Classic Instrument, MP Biomedicals, Santa Ana, CA, USA) in excess of TRI Reagent® (Sigma-Aldrich, Saint Louis, Mo, USA), and subsequently RNA was isolated according to the manufacturer's instructions. RNA concentration was measured with a DS-11 microvolume spectrophotometer (DeNovix, Wilmington, DE, USA). RNA integrity was determined by a Lab-on-a-Chip capillary electrophoresis system (RNA 6000 Nano Kit, 2100 Bioanalyzer Instruments and 2100 Expert Software, Agilent Genomics, Santa Clara, CA, USA). RNA Integrity Number (RIN) > 7 samples was used for further study. Residual DNA contamination was removed by DNase I (Worthington Biochemical Corporation, Lakewood, NJ, USA) treatment. After thermal inactivation of the enzyme, cDNA was synthesized from 200 ng of total RNA by SuperScript IV VILO Master Mix (Invitrogen, Thermo Fisher Scientific, Wilmington, USA); in parallel, no RT control reaction was conducted for each RNA sample. The STA (Single Tube TaqMan Gene Expression Assay) assays were as follows:* Ahsg* (Rn00563700_m1),* Fgf21* (Rn00590706_m1),* Rbp4* (Rn01451318_m1), and* Slc2a4* (Rn00562597_m1); reference STA assays were as follows:* Pum1* (Rn00982780_m1),* Rpl37a* (Rn02114291_s1), and* Tbp* (Rn01455648_m1); TaqMan Universal PCR Master Mix and ViiA 7 Real-Time PCR System (Thermo Fisher Scientific, Waltham, MA USA) were used for quantitative real-time PCR, which was performed in the 96-well format with an epMotion 5070 liquid handling workstation (Eppendorf, Hamburg, Germany). Four microliters of diluted (1:80) cDNA or no RT control sample was used in each PCR reaction; all reactions were performed in triplicate according to the original STA assay manufacturer's protocol, with a minor modification (10 *μ*l reaction volume). In the STA, no PCR amplification was detected for the no RT control samples before cycle number 40. Gene expression was determined with the ΔΔCq method. Normalisation was performed to a reference index obtained by calculating the geometric mean of reference gene (*Pum1*,* Rpl37a*, and* Tbp*) expression. The fold change of analyzed gene expression level was calculated as a difference between normalised values obtained from the same animal before and after surgery.

### 2.5. Statistical Analysis

Statistical analysis was performed using STATISTICA 12.5 PL (StatSoft, Cracow, Poland). Statistical significance was set at a* p* value below 0.05. All tests were two-tailed. Interval data were expressed as mean value ± standard deviation in the case of normal distribution or as median/lower–upper quartile range in the case of data with skewed or nonnormal distribution. Distribution of variables was evaluated by the Shapiro-Wilk test and the quantile-quantile plot- homogeneity of variances was assessed by the Levene test. For comparison of data, the two-way parametric ANOVA with post hoc contrast analysis or nonparametric Kruskal-Wallis test or the U Mann-Whitney test were used. In case of skewed data distribution, logarithmic transformation was done before analysis.

## 3. Results

The results of body weight change after DJOS and SHAM surgery in all experimental groups were presented in our previous study [19]. Briefly, DJOS surgery was shown to have very little impact on body weight reduction. In the DJOS groups, the body weight had no negative effect on insulin levels, glucose tolerance, or liver fat deposition. After DJOS, even though the body weight increased, the amelioration of glucose tolerance was reached for the HF/HF and CD/CD groups, but not for the mixed diet groups (HF/CD and CD/HF). Changes in diet after surgery influenced the glucose stimulated insulin secretion. After DJOS surgery, the glucose tolerance was ameliorated in both the HF/HF and CD/CD groups, but not in the groups of animals whose diet was changed from a HF to a CD and from a CD to a HF. The glucose amelioration in HF/HF subjects was found to be comparable with the control group, which can be an effect of ketogenic properties of a HF.

Table 1 shows the measured plasma concentrations of RBP4, fetuin-A, and FGF21 and the* Rbp4*,* fetuin-A (Ahsg)*, and* Fgf21* gene expression in the liver of animals that had been subjected to DJOS and SHAM surgery. For most of the analyzed hepatokines, their plasma concentrations were higher after DJOS surgery when a comparison between surgery and type of diet used was conducted. Table 1 also shows differences in gene expression of* Rbp4*,* Ahsg*, and* Fgf21* in the liver tissues of animals after both types of surgery. Significant differences in gene expressions were deduced from two-way ANOVA analysis between type of surgery, groups, and interaction between group and operation type.

When the two-way analysis of variance showed that one of the main analyzed factors is statistically significant and when also, but not necessarily, interaction between two main factors occurs, then contrast analysis can be performed. It means that we can compare each subclass of the first factor between groups defined by the first factor (p value for comparisons between types of operation, SHAM and DJOS) and each subclass of the second factor between groups defined by the first factor (p value for comparisons between diets, i.e., HF/HF, HF/CD, CD/HF, and CD/CD). Multiple comparisons in contrast analysis of hepatokines plasma levels in DJOS and SHAM operated groups in relation to diet used before and after surgery are presented in Table 2. Column one shows a comparison between DJOS and SHAM surgery associated with different diets, column two shows comparisons between dietary groups of DJOS animals, and column three shows comparisons between dietary groups of SHAM animals.

### 3.1. RBP4 Plasma Levels

#### 3.1.1. DJOS vs. SHAM Surgery

**Figure 2:**
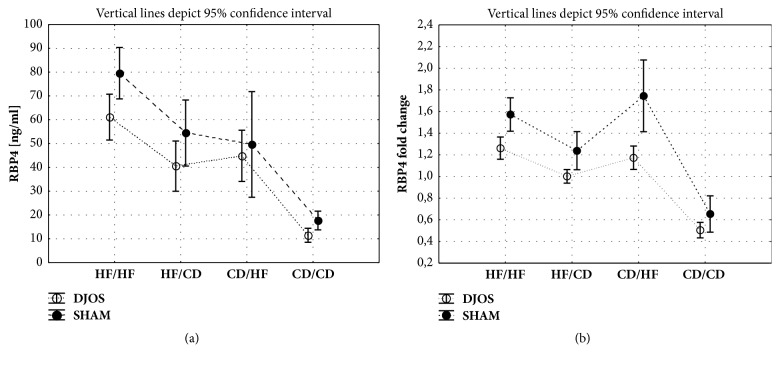
(a) Mean values of RBP4 plasma levels in groups subjected to different dietary patterns, according to the DJOS and SHAM operation type. Statistical significance was set at* p* < 0.05. Abbreviations: DJOS: duodenal-jejunal omega switch surgery; HF: high-fat diet; CD: control diet; HF/HF, CD/HF, HF/CD, and CD/CD: type of diet 8 weeks before/8 weeks after surgery. (b) Mean values of* Rbp4* fold change between groups subjected to different dietary patterns, according to the DJOS and SHAM operation type.

DJOS surgery had a significant reductive impact on the RBP4 plasma levels in the groups of animals kept on HF/HF and HF/CD, when compared to SHAM surgery (Figure 2(a), Table 1 (general comparison), and Table 2 (multiple comparisons)).

#### 3.1.2. DJOS Surgery

Plasma concentrations of RBP4 were significantly higher in HF/HF group when compared to other studied groups (Figure 2(a); Tables 1 and 2). In the groups maintained on mixed diet (HF/CD and CD/HF) the level of RBP4 was significantly higher in comparison to CD/CD group (Figure 2(a), Table 2).

#### 3.1.3. SHAM Surgery

The same trend in RBP4 plasma levels as for DJOS surgery was observed for SHAM operated animals (Figure 2(a), Table 2).

### 3.2. Rbp4 Expression

#### 3.2.1. DJOS vs. SHAM Surgery

Type of surgery, diet, and interaction between group and operation type had significant impact on the* Rbp4* liver expression (Table 1). Significantly higher expression of* Rbp4* was observed in animals maintained on HF/HF and CD/HF after SHAM surgery when compared to DJOS (Figure 2(b); Tables 1 and 2).

#### 3.2.2. DJOS Surgery

A higher expression of* Rbp4* gene was observed in the groups of animals kept on HF/HF, HF/CD, and CD/HF as compared with the CD/CD group (Figure 2(b); Tables 1 and 2).* Rbp4* gene expression was also significantly stronger in the HF/HF than in the HF/CD group (Figure 2(b); Tables 1 and 2).

#### 3.2.3. SHAM Surgery

Similar differences in* Rbp4* gene expression as in the DJOS surgery were observed in SHAM operated animals exposed to HF/HF and mixed diet before and after surgery as compared with the CD/CD group (Figure 2(b); Tables 1 and 2). Significant differences were also observed between the HF/CD and HF/HF as well as the HF/CD and CD/HF groups.

### 3.3. Fetuin-A Plasma Levels

#### 3.3.1. DJOS vs. SHAM Surgery

**Figure 3:**
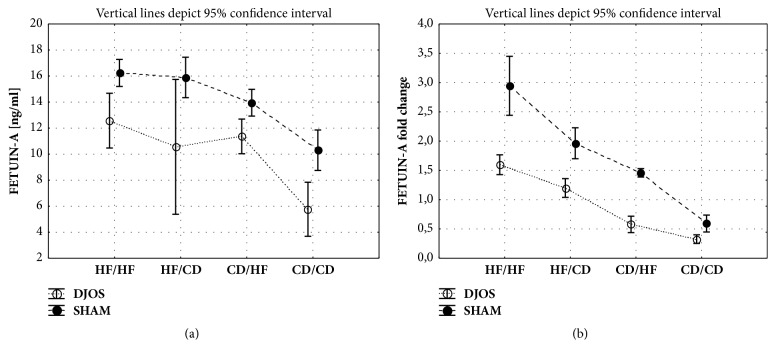
(a) Mean values of fetuin-A plasma levels in groups of different dietary patterns, according to the DJOS and SHAM operation type. Statistical significance was set at* p* < 0.05. Abbreviations: DJOS: duodenal-jejunal omega switch surgery; HF: high-fat diet; CD: control diet; HF/HF, CD/HF, HF/CD, and CD/CD: type of diet 8 weeks before/8 weeks after surgery. (b) Mean values of* Ahsg* (fetuin-A) fold change between groups subjected to different dietary patterns, according to the DJOS and SHAM operation type.

Significant differences in fetuin-A plasma concentrations between DJOS and SHAM groups were found for HF/HF, HF/CD, and CD/CD groups (Figure 3(a), Tables 1 and 2). In those groups fetuin-A levels were significantly lower in animals which underwent DJOS surgery as compared to SHAM surgery.

#### 3.3.2. DJOS Surgery

The lowest plasma level of fetuin-A was observed in the CD/CD group in comparison to the HF/HF group and mixed diets (HF/CD, CD/HF) (Figure 3(a), Table 2).

#### 3.3.3. SHAM Surgery

In the SHAM operated animals, the CD/CD group showed a significantly lower fetuin-A plasma level as compared with other studied groups (Figure 3(a), Table 2).

### 3.4. Fetuin-A Gene Expression (Ahsg)

#### 3.4.1. DJOS vs. SHAM Surgery

The DJOS and SHAM surgery, diet, and interaction between group and operation type had significant impact on the* Ahsg* liver expression (Table 1). The relative expression of the* fetuin-A* gene in the liver of DJOS operated animals was significantly lower compared with SHAM operated groups (Figure 3(b), Tables 1 and 2).

#### 3.4.2. DJOS Surgery

The* Ahsg *expression in DJOS operated animals was higher in the group kept on a HF diet before and after the surgery as compared with other groups (Figure 3(b), Tables 1 and 2). Mixed type of diet before and after the surgery (HF/CD) showed significantly higher relative* Ahsg* expression when compared to the CD/HF and CD/CD groups (Figure 3(b), Tables 1 and 2).

#### 3.4.3. SHAM Surgery

The* Ahsg* mRNA levels significantly varied among all the studied groups after SHAM surgery. Significantly higher values were observed in the HF/HF group when compared to the HF/CD, CD/HF, and CD/CD animals (Figure 3(b), Tables 1 and 2). The mixed dietary patterns (HF/CD and CD/HF) stimulated an enhanced expression of* Ahsg *when compared to the CD/CD group (Figure 3(b), Tables 1 and 2). For the mixed diet groups,* Ahsg *expression in the liver tissue was shown to be significantly higher in the HF/CD group than the CD/HF animals (Figure 3(b), Tables 1 and 2).

### 3.5. FGF 21 Plasma Levels

#### 3.5.1. DJOS vs. SHAM

**Figure 4:**
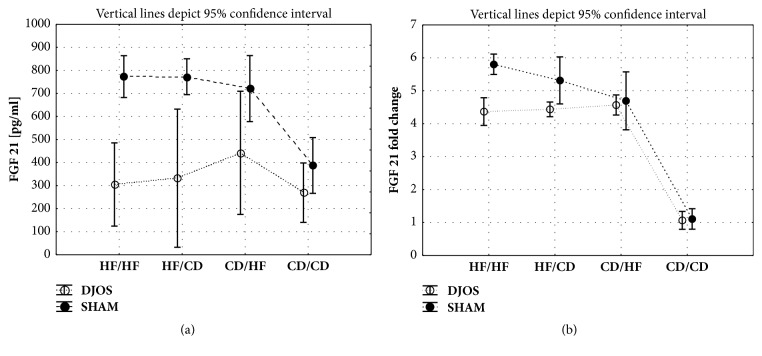
(a) Mean values of FGF 21 plasma levels in groups of diet, according to the DJOS and SHAM operation type. Statistical significance was set at* p* < 0.05. Abbreviations: DJOS: duodenal-jejunal omega switch surgery; HF: high-fat diet; CD: control diet; HF/HF, CD/HF, HF/CD, and CD/CD: type of diet 8 weeks before/8 weeks after surgery. (b) Mean values of* Fgf21* fold change in groups of different dietary patterns, according to the DJOS and SHAM operation type.

In DJOS and SHAM groups an interaction between group and operation type was observed. Significant differences between DJOS and SHAM type of surgery independently to the diet, in the analyzed groups, were observed (Table 1). Rats which underwent DJOS surgery showed a significantly lower plasma concentration of FGF 21 than SHAM operated animals in the HF/HF, HF/CD, and CD/HF diet groups (Figure 4(a), Tables 1 and 2).

#### 3.5.2. DJOS Surgery

There were no significant differences in FGF 21 plasma concentration between the DJOS operated animals (Figure 4(a), Tables 1 and 2).

#### 3.5.3. SHAM Surgery

Among SHAM operated animals, the HF/HF, HF/CD, and CD/HF diet groups exhibited similar FGF 21 levels as the DJOS operated rats; these concentrations were significantly higher than in the CD/CD group (Figure 4(a), Table 2).

### 3.6. Fgf21 Gene Expression

#### 3.6.1. DJOS vs. SHAM Surgery

*Fgf21* gene expression was significantly different in relation to DJOS and SHAM type of procedure (Table 1). Diet and interaction between group and operation type had significant impact on the* Fgf21* liver expression (Table 1). Comparing DJOS and SHAM type of surgery, the relative* Fgf21* gene expressions were significantly lower in the HF/HF and HF/CD groups after DJOS when compared to SHAM (Figure 4(b), Tables 1 and 2).

#### 3.6.2. DJOS Surgery

The* Fgf21* gene expressions in the liver of animals kept on HF/HF and mixed dietary patterns (HF/CD and CD/HF) were significantly higher as compared to the CD/CD group (Figure 4(b), Tables 1 and 2).

#### 3.6.3. SHAM Surgery

The CD/CD group showed significantly lower* Fgf21* gene expression in comparison with the HF/HF, HF/CD, and CD/HF groups. Significant differences were also observed between the HF/HF vs. CD/HF and HF/CD vs. CD/HF groups (Figure 4(b), Tables 1 and 2).

## 4. Discussion

To better understand the metabolic changes under the conditions of obesity, we have here analyzed selected hepatokines in relation to different dietary patterns in combination with duodenal exclusion. In this study, we present the effects of HF and CD in combination with DJOS on plasma concentrations of RBP4, fetuin-A, and FGF21 as well as their mRNA expression in the liver. We can state the following: (i) Both diet and surgery strongly influenced plasma levels of selected hepatokines. A reducing impact of DJOS surgery on plasma levels of RBP4, fetuin-A, and FGF21 was detected. (ii) Regardless of surgery, the type of dietary pattern applied before and after the surgery had an impact on RBP4, fetuin-A, and FGF21 plasma levels. Taking into consideration the type of diet, comparing DJOS and SHAM, similar changes in RBP 4, fetuin-A, and FGF21 plasma levels were observed. (iii) The highest levels of RBP 4 and fetuin-A, but not FGF21, were detected in the plasma of animals handled on HF before and after the surgery in comparison to other groups; thus HF has a reductive impact on the beneficial effect of DJOS surgery. (iv) The lowest levels of RBP4 and fetuin-A were characteristic for animals kept on a control diet before and after the surgery. (v) DJOS surgery had a significant reductive impact on relative gene expression of* Rbp4*,* Ahsg*, and* Fgf21*, when compared to SHAM operated animals. (vi) Regardless of the type of surgery, the type of dietary pattern applied before and after the surgery influenced the relative gene expression of selected hepatokines. HF used before and/or after the surgery led to upregulation of* Rbp4*,* Ahsg*, and* FGF21* relative gene expression when compared to the control.

DJOS surgery was shown to significantly decrease the RBP4 plasma levels and* Rbp4* expression level in the liver of rats, regardless of diet used before or after surgery as compared with SHAM operated animals. Nevertheless, regardless of the type of surgery, HF or mixed type of dietary pattern stimulated* Rbp4* expression and increased plasma concentration of RBP4 when compared to the control diet. RBP4 is synthesized by hepatocytes and—to a lesser extent—by adipocytes. It is responsible for retinol transport from the liver to peripheral tissues [21]. Overexpression of* Rbp4* in adipocytes is reported in the conditions of insulin resistance, metabolic syndrome, and obesity [7]. Our results are consistent with other observations obtained on animals models, which show that injection of RBP4 into mice or transgenic overexpression of* Rbp4 *in mice leads to impaired insulin signalling in the skeletal muscle tissue and influences the activity of the gluconeogenic enzymes in the liver [22]. Wang et al. observed upregulation of the* Rbp4* mRNA expression in the liver and increased RBP4 plasma concentrations in Wistar rats after 4 and 8 weeks of high cholesterol diet, in comparison with control group, and downregulation of* Rbp4* mRNA expression after 12 weeks of high cholesterol diet [23]. This is probably the effect of rising RBP4 plasma concentration, which may reduce the* Rbp4* mRNA liver expression. Stefan et al. observed that low hepatic fat content is correlated with decreased RBP4 plasma levels and high insulin clearance [24]. In our previous study, a reduced content of fat in the liver after DJOS surgery was observed in animals kept on HF/HF diet in comparison to SHAM operated animals [19]. Based on our results, we can suggest a strong relationship between the plasma concentration of RBP4 and hepatic fat accumulation. We observed a significant impact of DOJS surgery on the RBP4 plasma levels and hepatic* Rbp4* gene expression in all dietary patterns applied. Similar results for different types of bariatric surgery, but without involvement of dietary patterns, were noticed after RYGB in proteomic study, where RBP4 was reduced up to 72% [25, 26].

Fetuin-A acts as an endogenous ligand for TLR4 which stimulates adipose tissue inflammation and regulates insulin sensitivity [27, 28]. The role of fetuin-A in the pathophysiology of insulin resistance in rodents was proved by studies conducted on* Ahsg* knockout mice (KO) [29]. The fetuin-A KO animals showed increased insulin signalling, sensitivity, and resistance to weight gain to the adipogenic effect of the HF. Remarkably, KO mice fed with HF remained lean, with body weight comparable to the control group [29]. Human studies show that fetuin-A plasma levels correlate positively with the liver fat accumulation in nondiabetic individuals, while* Ahsg* mRNA expression is upregulated in hepatic dysfunction [30]. In a rat model of diet-induced obesity, which commonly displays fatty liver, an upregulation in* Ahsg* mRNA expression was also observed in the liver [12]. As mentioned above, a reduced fat content in the liver of HF/HF DJOS operated animals was observed as compared with HF/HF SHAM operated animals [19]. In that study, we have shown that the fetuin-A plasma level and the* Ahsg* mRNA expression in the liver are lower in animals after DJOS surgery as compared with SHAM groups, regardless of the dietary pattern. Any use of HF such as HF/HF, mixed HF/CD, and CD/HF stimulated the fetuin-A plasma concentrations and triggered the mRNA expression in the liver tissue as compared with the control diet. T2DM, as a comorbidity of obesity, is a very complex disease, where the gene expression and development are triggered/regulated by a combination of genetic, epigenetic, environmental, and lifestyle factors, including diet [31]. Despite the fact that T2DM is known to be a highly heritable disease, only 10% to 15% of the heritability of the disease is explained by known genetic mechanisms [32].

It is known that FGF 21 is one of the main factors involved in the pathogenesis and therapeutic mechanisms of NAFLD [33]. FGF21 is upregulated by the nutrient sensor, SIRT1, which ameliorates hepatic IR in diabetic obese mice through suppression of mTORC1 [34]. The studies conducted* in vivo* and* in vitro* prove that FGF21 inhibits nutrient- and hormonal-induced hepatic mTORC1 activity. During starvation FGF21 may be induced by activation of TORC1 stimulated by TSC1 deficiency. Under this conditions FGF21 acts in an autocrine fashion regulating liver fatty acid metabolism, promoting glucose utilization, and controlling peripheral glucose homeostasis by stimulating hepatic glycogen storage [34, 35]. Diet-induced obese, genetically obese, diabetic db/db and ob/ob mice were shown to have increased endogenous levels of FGF21 [36, 37]. Also,* Fgf21* expression was increased in the liver of ob/ob mice when compared to lean littermates [38]. These data led to the hypothesis that increased plasma FGF21 levels may be a prognostic factor of metabolic syndrome and T2DM and that these may be states of relative FGF21-resistance [37]. In the present study, we show that the FGF21 plasma level was lowered after DJOS surgery in comparison with SHAM groups, even after long-term consumption of HF. Similar results, but not related to the diet, were observed in human studies after laparoscopic sleeve gastrectomy, where FGF21 plasma levels in obese subjects were significantly higher in comparison to the control and decreased 12 and 24 months after the surgery [39]. Here, the DJOS surgery reduced the possible deleterious impact of HF, which was observed in SHAM operated animals in the HF/HF, HF/CD, and CD/HF groups, where FGF21 plasma levels and* Fgf21* gene expression were upregulated. Again the lowest values of this protein were observed after DJOS surgery in the CD/CD group. Other studies have shown that a very low-calorie diet applied for two weeks increased the FGF21 plasma levels in obese patients with T2DM [40]. Food restriction and low caloric diet appear to enhance hepatic FGF21 production, which helps to facilitate the physiological response to energy deficiency by reduction of gluconeogenesis and increase of hepatic ketogenesis via FGF21 [41]. Nonetheless, the plasma concentration of FGF21 is also known to be elevated under conditions of metabolic dysfunction, such as obesity [40]. Our present results may explain an influence of DJOS surgery on the normalisation of the FGF21 sensitivity, which has been reported to be highly reduced under obesity conditions [35]. Downregulation of the* Fgf21* mRNA expression after DJOS surgery may also suggest amelioration in the liver metabolic functions, since the liver may be responsible for the rise of the plasma FGF21 levels during overfeeding.

## 5. Conclusions

The efficiency of the DJOS surgery, measured by hepatokines' plasma levels and their gene expressions in the liver, depends on the type of diet applied before and after the surgery. HF, despite the combination (HF/HF, HF/CD, and CD/HF) used before and/or after surgery, showed a deleterious impact, reducing the effect of the surgery. These data demonstrate that manipulation of dietary patterns can lead to marked improvements in metabolic profile after DJOS surgery.

## Figures and Tables

**Table 1:** Plasma levels of RBP4, fetuin-A, FGF21, and fold change of *Rbp4, FetuinA (Ahsg), *and* Fgf21* in liver tissue 8 weeks after DJOS (1^st^ column) and SHAM (2^nd^ column) surgery, subjected to 16 weeks of different dietary patterns, and intergroup comparison between DJOS and SHAM study groups (3^rd^ column) using descriptive statistics and results of two-way analysis of variance.

	**DJOS**				**SHAM**				**p ANOVA**		
**Parameter**	**HF/HF**	**HF/CD**	**CD/HF**	**CD/CD**	**HF/HF**	**HF/CD**	**CD/HF**	**CD/CD**	**Group**	**Op.**	**Int.**
**Plasma levels**											

**RBP-4 [ng/mL]**	61.1 ± 10.4	40.5 ± 10.0	44.8 ± 10.2	11.5 ± 3.2	79.6 ± 11.7	54.4 ± 12.2	49.6 ± 21.1	17.7 ± 4.2	**<0.001**	**<0.01**	0.377
**Fetuin-A [ng/mL]**	12.6 ± 2.3	10.6 ± 4.9	11.4 ± 1.3	5.8 ± 2.2	16.2 ± 1.1	15.9 ± 1.5	14.0 ± 1.0	10.3 ± 1.7	**<0.001**	**<0.001**	0.498
**FGF 21 [ng/mL]**	304.8 ± 195.3	332.2 ± 285.5	442.3 ± 254.8	269.3 ± 139.5	772.9 ± 98.3	772.7 ± 74.1	721.1 ± 136.4	387.5 ± 130.8	**<0.01**	**<0.001**	**<0.05**

**Fold change**											

**RBP-4**	1.26 ± 0.12	1.00 ± 0.08	1.17 ± 0.14	0.50 ± 0.09	1.57 ± 0.17	1.24 ± 0.23	1.74 ± 0.43	0.65 ± 0.22	**<0.001**	**<0.001**	**<0.05**
**Fetuin-A *(Ahsg)***	1.60 ± 0.22	1.20 ± 0.21	0.58 ± 0.18	0.33 ± 0.10	2.94 ± 0.60	1.96 ± 0.34	1.46 ± 0.09	0.59 ± 0.19	**<0.001**	**<0.001**	**<0.001**
**FGF 21**	4.37 ± 0.55	4.44 ± 0.29	4.57 ± 0.40	1.07 ± 0.35	5.80 ± 0.40	5.31 ± 0.93	4.69 ± 1.14	1.11 ± 0.40	**<0.001**	**<0.001**	**<0.01**

Statistical significance was set at a *p* < 0.05. Abbreviations: RBP4: retinol binding protein 4; FGF21: fibroblast growth factor 21. DJOS: duodenal-jejunal omega switch surgery; HF: high-fat diet; CD: control diet; HF/HF, CD/HF, HF/CD, and CD/CD: type of diet 8 weeks before/8 weeks after surgery; Op.: operation type; Int.: interaction between group and operation type. Mean ± standard deviation or median (lower – upper quartile).

**Table 2:** Multiple comparisons in contrast analysis of RBP4, fetuin-A (*Ahsg*), FGF21 plasma levels, and relative gene expression in liver tissue. Column 1: intergroup comparisons between HF/HF, CD/HF, HF/CD, and CD/CD groups of DJOS and SHAM. Column 2: intragroup comparisons between HF/HF, CD/HF, HF/CD, and CD/CD groups after DJOS surgery. Column 3: intragroup comparisons between HF/HF, CD/HF, HF/CD, and CD/CD groups after SHAM surgery.

**Post-hoc**	**DJOS vs SHAM**				**DJOS**						**SHAM**					
	**1: HF/HF**	**2: HF/CD**	**3: CD/HF**	**4: CD/CD**	**1 vs2**	**1 vs 3**	**1 vs 4**	**2 vs 3**	**2 vs 4**	**3 vs 4**	**1 vs 2**	**1 vs 3**	**1 vs 4**	**2 vs 3**	**2 vs 4**	**3 vs 4**
**Plasma levels**																

**RBP-4 [ng/mL]**	**<0.01**	**<0.01**	0.473	0.318	**<0.01**	**<0.05**	**<0.001**	0.517	**<0.001**	**<0.001**	**<0.001**	**<0.001**	**<0.001**	0.471	**<0.001**	**<0.001**
**Fetuin-A [ng/mL]**	**<0.01**	**<0.001**	0.057	**<0.001**	0.121	0.349	**<0.001**	0.545	**<0.001**	**<0.001**	0.786	0.080	**<0.001**	0.150	**<0.001**	**<0.01**
**FGF 21 [ng/mL]**	**<0.001**	**<0.001**	**<0.01**	0.215	0.780	0.167	0.708	0.284	0.523	0.084	0.998	0.598	**<0.001**	0.613	**<0.001**	**<0.01**

**Fold Change**																

**RBP-4**	**<0.01**	0.023	**<0.001**	0.149	**<0.05**	0.402	**<0.001**	0.096	**<0.001**	**<0.001**	**<0.01**	0.118	**<0.001**	**<0.001**	**<0.001**	**<0.001**
**Fetuin-A *(Ahsg)***	**<0.001**	**<0.001**	**<0.001**	**<0.05**	**<0.05**	**<0.01**	**<0.001**	**<0.001**	**<0.001**	0.062	**<0.001**	**<0.001**	**<0.001**	**<0.001**	**<0.001**	**<0.001**
**FGF 21**	**<0.001**	**<0.01**	0.675	0.886	0.820	0.502	**<0.001**	0.656	**<0.001**	**<0.001**	0.103	**<0.001**	**<0.001**	**<0.05**	**<0.001**	**<0.001**

Post hoc analysis: statistical significance was set at a *p* < 0.05. Abbreviations: RBP4: retinol binding protein 4; FGF21: fibroblast growth factor 21; DJOS: duodenal-jejunal omega switch surgery; HF: high-fat diet; CD: control diet; HF/HF, CD/HF, HF/CD, and CD/CD: type of diet 8 weeks before/8 weeks after surgery.

## Data Availability

The data used to support the findings of this study are available from the corresponding author upon request.

## Supplementary Materials

Supplementary Materials The composition of the diets used in the experiment.
