# Supplementary material for: Changes in Liver Gene Expression and Plasma Concentration of Rbp4, Fetuin-A, and Fgf21 in Sprague-Dawley Rats Subjected to Different Dietary Interventions and Bariatric Surgery
Source: Biomed Res Int. 2018 Aug 16;2018:3472190. doi: 10.1155/2018/3472190 (PMC6126060; doi:10.1155/2018/3472190)
Supplement: Supplementary Materials — The composition of the diets used in the experiment. [file 3472190.f1.pdf]

Supplementary Material

Table 1. The composition of the diets used in the experiment.

**CONTROL DIET**<br><br>**Mouse and rat, breeding**<br><br>**Standart diet**<br><br>**Producer:** Provimi Kliba AG, Kaiseraugst, Switzerland		**HIGH FAT DIET**<br><br>**ssniff® EF R/M acc. D12451 (II) mod. ***<br><br>**Experimental diet for rats and mice, high fat content (tallow) / DIO series**<br><br>**Producer:** Ssniff Spezialdiäten GmbH, Soest, Germany	
**Crude Nutrients**			
Dry matter	88.1 %	Dry matter	96.9 %
Crude protein	24.0 %	Crude protein	(N x 6.25) 22.5%
Crude fat	4.9 %	Crude fat	23.1 %
Crude fiber	4.7 %	Crude fiber	5.7 %
Crude ash	7.0 %	Crude ash	5.9 %
N free extracts	47.5 %	N free extracts	39.8 %
Gross energy	16.5 MJ/kg	Gross energy	22.1 MJ/kg
Metabol. energy	13.5 MJ/kg	Metabol. energy	19.2 MJ/kg
Starch	29.0 %	Starch	8.6 %
		Sugar / Dextrines	29.4 %
**Amino acids**			
Arginine	1.51 %	Arginine	0.82 %
Lysine	1.36 %	Lysine	1.85 %
Methionine	0.45 %	Methionine	0.78 %
Methionine + cystine	0.82 %	Met+Cysteine	1.13 %
Tryptophan	0.30 %	Tryptophan	0.29 %
Threonine	0.90 %	Threonine	1.00 %
		Histidine	0.71 %
		Serine	1.34 %

		Alanine	0.74 %
		Proline	2.59 %
		Aspartic acid	1.68 %
		Glutamic acid	5.08 %
		Glycine	0.47 %
		Phe+Tyr	2.41 %
		Phenylalanine	1.21 %
		Leucine	2.22 %
		Isoleucine	1.18 %
		Valine	1.54 %
		Cystine	0.35 %
**Major mineral elements**			
Calcium	1.20 %	Calcium	1.05 %
Phosphorus	0.83 %	Phosphorus	0.69 %
Magnesium	0.22 %	Magnesium	0.15 %
Sodium	0.23 %	Sodium	0.20 %
Potassium	0.95 %	Potassium	0.72 %
Chlorine	0.41 %		
**Trace elements**			
Iron	250 mg/kg	Iron	122 mg/kg
Zinc	64 mg/kg	Zinc	50 mg/kg
Copper	15 mg/kg	Copper	10 mg/kg
Iodine	1.2 mg/kg	Iodine	0.85 mg/kg
Manganese	55 mg/kg	Manganese	72 mg/kg
Selenium	0.3 mg/kg	Selenium	0.12 mg/kg
**Vitamins**			
Vitamin A	12 000 IU/kg	Vitamin A	15 000 IU/kg
Vitamin D3	1'000 IU/kg	Vitamin D3	1.500 IU/kg
Vitamin E	95 mg/kg	Vitamin E	150 mg/kg
Vitamin K3	4 mg/kg	Vitamin K (as menadione)	20 mg/kg
Vitamin B1	22 mg/kg	Thiamin (B1)	16 mg/kg

Vitamin B2	13 mg/kg	Riboflavin (B2)	16 mg/kg
Vitamin B6	10 mg/kg	Pyridoxine (B6)	18 mg/kg
Vitamin B12	0.05 mg/kg	Cobalamin (B12)	0.030 mg/kg
Nicotinic acid	70 mg/kg	Nicotinic acid	45 mg/kg
Pantothenic	30 mg/kg	Pantothenic acid	55 mg/kg
Folic acid	3 mg/kg	Folic acid	19 mg/kg
Biotin	0.26 mg/kg	Biotin	0.305 mg/kg
Choline	1'800 mg/kg	Choline-Chloride	1,050 mg
Vitamin C	36 mg/kg	Vitamin C	30 mg/kg
		Inositol	80 mg
**Fatty acids**			
		C 8:0	—
		C10:0	—
		C12:0	0.02 %
		C14:0	0.69 %
		C16:0	5.39 %
		C16:1	0.52 %
		C17:0	0.25 %
		C18:0	3.75 %
		C18:1	8.17 %
		C18:2	1.84 %
		C18:3	0.25 %
		C20:0	0.03 %
		C20:1	0.01 %
		C20:4	0.05 %
		C20:5	—
		C22:6	—
		Cholesterol (original content)	194 mg/kg
